# Test Pattern Design for Plasma Induced Damage on Inter-Metal Dielectric in FinFET Cu BEOL Processes

**DOI:** 10.1186/s11671-020-03328-7

**Published:** 2020-05-01

**Authors:** Chi Su, Yi-Pei Tsai, Chrong-Jung Lin, Ya-Chin King

**Affiliations:** Institute of Electronics Engineering, National Tsing Hua University, Hsinchu, 300 Taiwan

**Keywords:** Plasma-induced damage, Advanced FinFET technology, Back-end of line, Inter-metal dielectric

## Abstract

High-density interconnects, enabled by advanced CMOS Cu BEOL technologies, lead to closely placed metals layers. High-aspect ratio metal lines require extensive plasma etching processes, which may cause reliability concerns on inter metal dielectric (IMD) layers. This study presents newly proposed test patterns for evaluating the effect of plasma-induced charging effect on the integrity of IMD between closely placed metal lines. Strong correlations between the plasma charging intensities and damages found in IMD layers are found and analyzed comprehensively.

## Introduction

Cu-based back-end of line (BEOL) processes have been used extensively as technologies migrate to sub-100 nm regime. Tightly packed interconnects consist of high-aspect ratio vias and metal lines are made possible by a series of plasma-enhanced etching processes [1–3]. It is well known that high-energy plasma treatments can lead to significant degradation and latent damages in transistors’ gate dielectric stacks, which are believed to be the main discharging path during process-induced charging events. To prevent reliability concerns on the gate dielectric quality, IC manufacturers typically provide designer rules and guidelines which limit the size and length of interconnect metal layers [4, 5]. With increased number of metal layers in the complex wiring systems, it is hard to avoid discharging path through inter metal dielectric film during process-induced charging. With the introduction of low-k material in BEOL processes [6, 7], alleviated worsen RC delay issues, the isolation films can be more susceptible to charging stresses [8, 9]. The damages resulting from plasma charging-induced stresses on compact interconnect structures responsible for realizing the intricate BEOL wirings can greatly affect the yield and reliability of advanced CMOS ICs. In this study, we incorporate previously reported the in situ PID recorders [10–12], with newly proposed differential test patterns for monitoring its possible IMD damage when additional discharging path become possible under advanced Cu BEOL processes. The plasma charging levels reported the recorder at different locations across wafer can be correlated with plasma charging damage on IMD films through the new test patterns. Stress-induced failure behaviors on the IMD structures can then be easily revealed through these test patterns which can be used in standard test structure for routine monitoring of new failure modes and possible IMD reliability concerns.

## Device Structure and Methodology

**Fig. 1:**
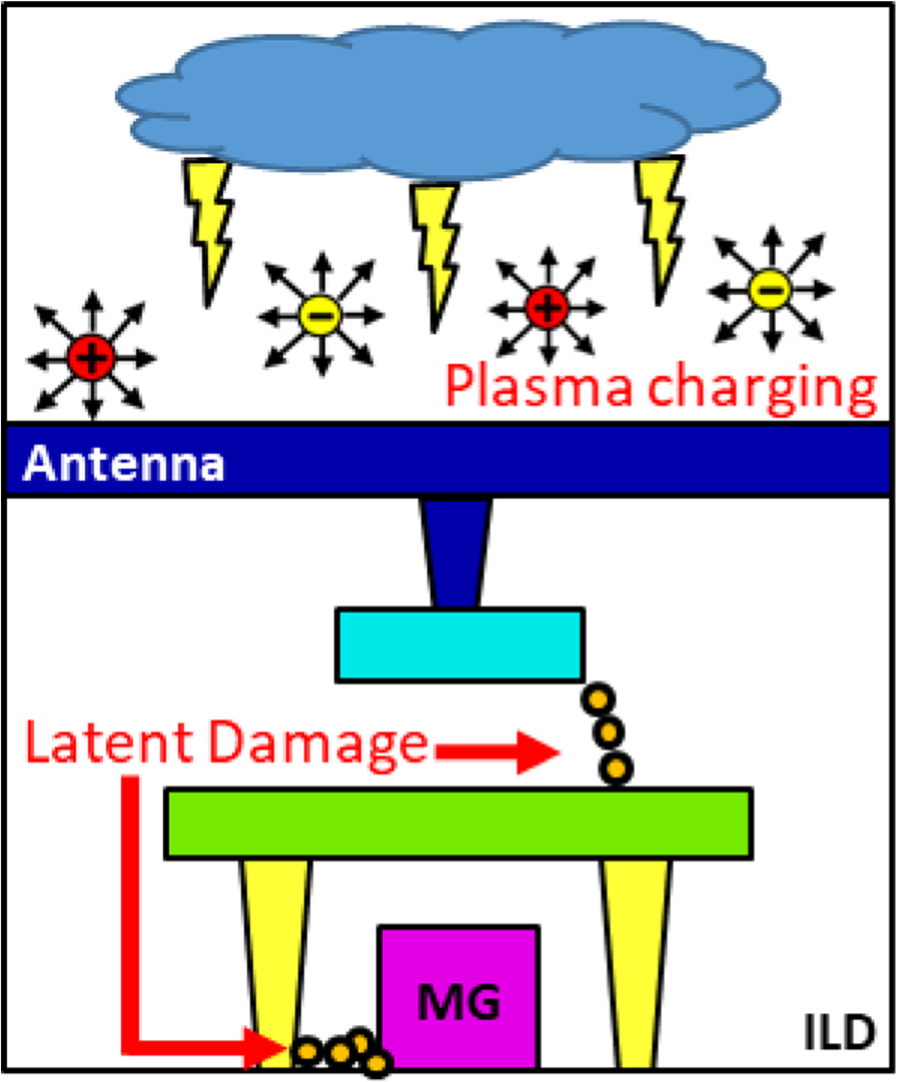
Plasma-induced damages in inter-metal dielectric layers between tightly packed interconnect patterns can link to latent degradation on its isolation integrity

Failure analysis results on FinFET-integrated circuits suggest that latent damages of increase trap states within inter-metal dielectric layers between two closely placed isolated interconnect structures may occur, as illustrated in Fig. 1. As the line width and spacing between via and metal interconnects reduce aggressively in advance CMOS BEOL processes, charges collected on large metal wires, serves as antennas, may discharge through IMD across metals and vias/gates and metal wires, resulting in high-field and/or high-current stresses on these dielectric films. By placing the in situ PID recorder [10–12] next to the two differential test patterns on each die, the reference plasma charging levels across a 12-inch wafer can first be established.

**Fig. 2:**
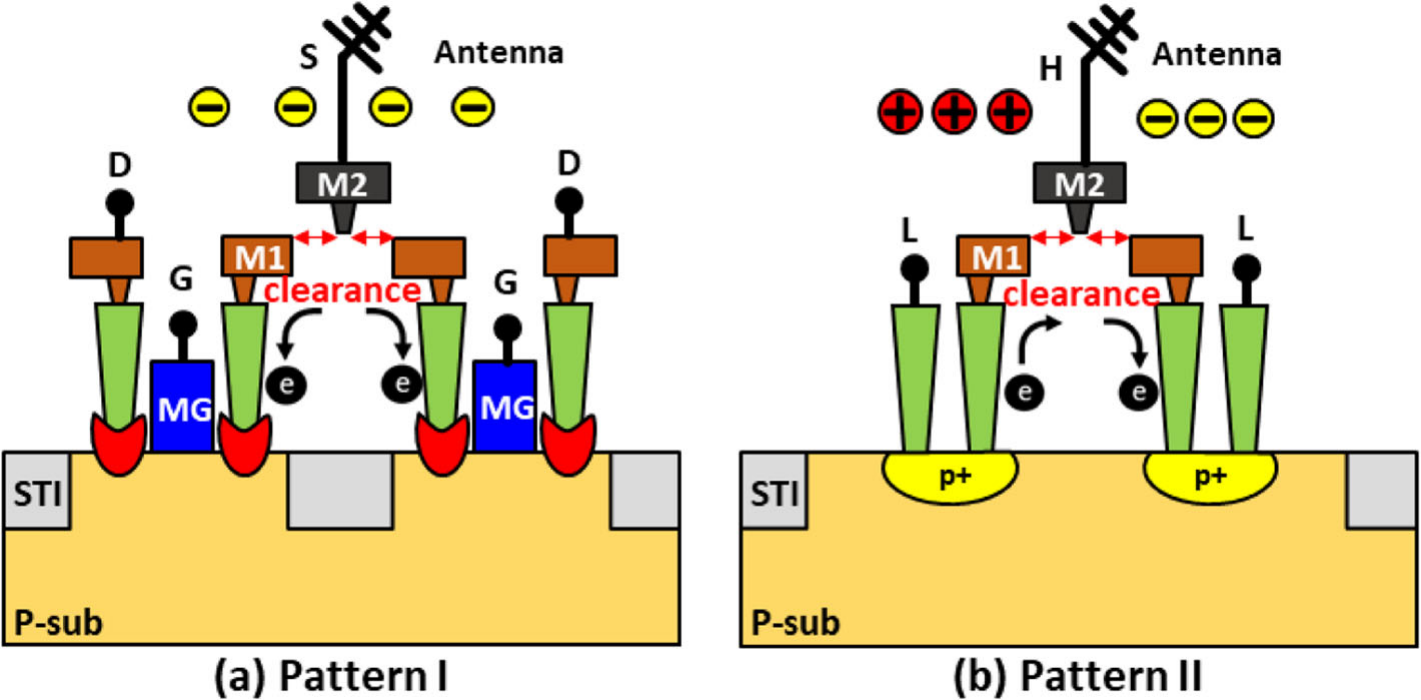
Illustrations of newly proposed differential test patterns designed to accentuate the effect of latent damage caused by plasma induced **a** negative and **b** bi-directional stresses, where the clearance between metal layer to set to 14 nm

To ensure that to the plasma charging current passing through the dielectric layer between metal layers, the M2 node on test pattern in Fig. 2 is isolated prior to the making of antenna structure. Two new differential test pattern designs aimed at increasing the possibility of causing the latent damage during plasma charging stresses are proposed and illustrated in Fig. 2a, b. The device with type I pattern will only experience high current stress when electrons are collected on its corresponding antenna. This is because the discharging current is rectified by the n+/p junction in series on the conductive pathway. Whereas, the device with type II pattern is subjected to stresses I both directions [13]. Namely, both ion charging and electron charging will be registered on this monitoring device. Consequently, samples in this study are made by the standard FinFET/Cu BEOL process in 16 nm technology node. The antenna structures on a PID recorder as well as the devices with type I and II patterns placed on each die are all designed with a large metal 3 structure. Both types of monitoring devices are designed with differential configuration, which can accentuate and subsequently minimizing the misalignments noise that might lead to fault readings on the PID effects.

## Experimental Results and Discussion

**Fig. 3:**
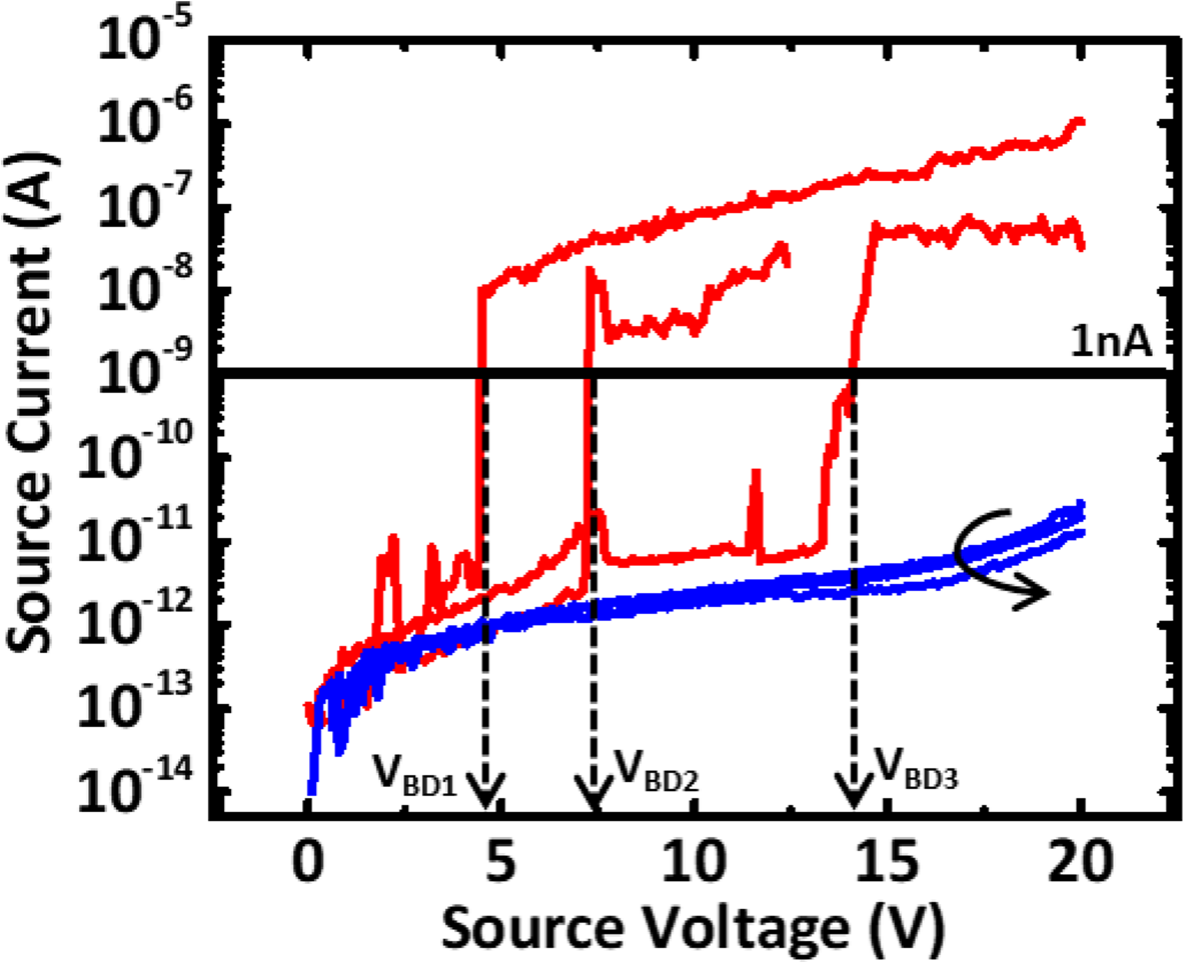
Leakage current measured on devices with differential test patterns connected to metal-3 antenna of 82,000 μm^2^. *V*_BD_ is defined as the voltage as the current reaches 1 nA for a via length of 32 nm

Data in Fig. 3 are obtained by applying a voltage sweep on metal 2 from 0–20 V at the sweep rate of 0.7 V/sec, while the breakdown voltage, and IMD leakage current on either side of a few different devices with test pattern I can then be obtained and compared. During making of the large M3 antenna of 82,000 μm^2^, plasma process is expected to induce charging of the antenna. The accumulated charge is expected to discharge through the pathway with the lowest resistance. Data reveals that some samples exhibit early dielectric breakdown, while IMD on other devices remains relatively intact with low leakage.

**Fig.4:**
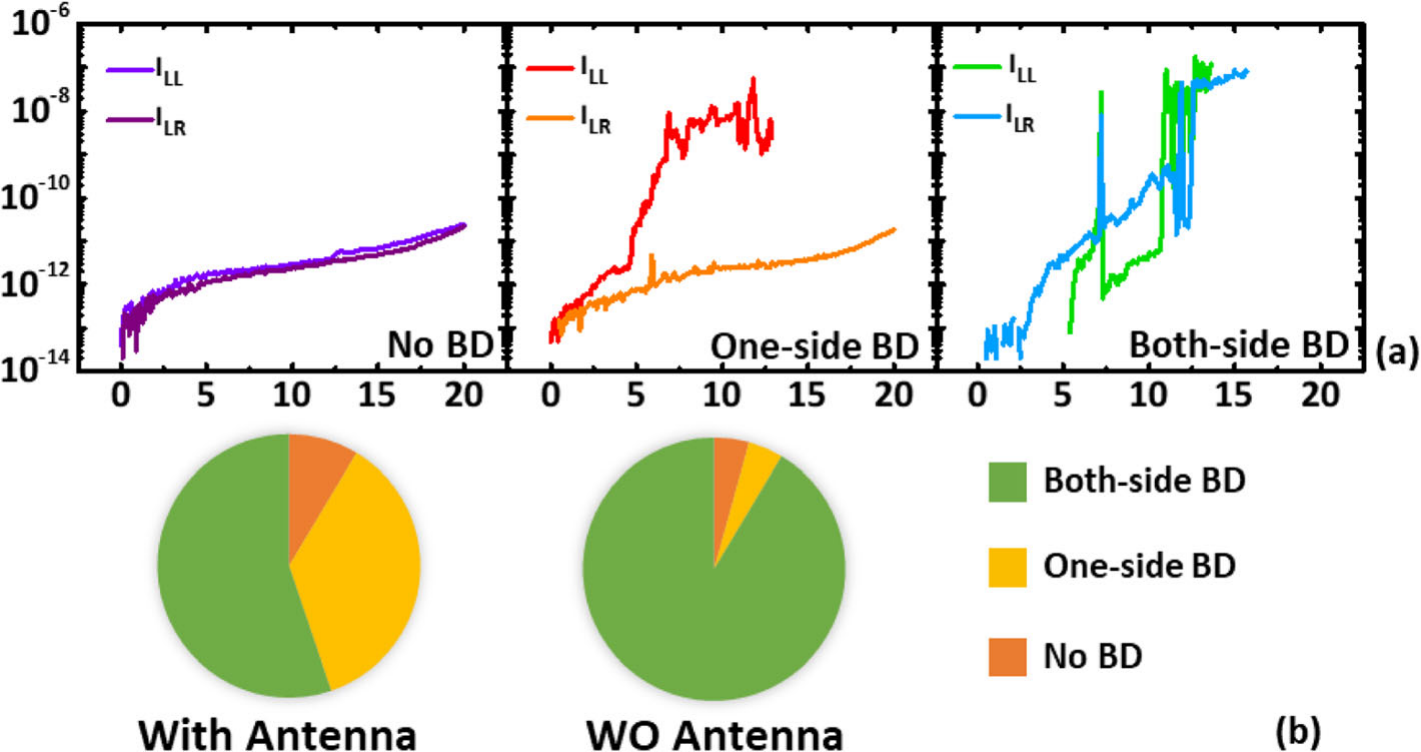
**a** Different types of breakdown characteristics on the test pairs across a wafer and **b** comparing the percentage of samples exhibiting distinctive types of breakdown characteristics on device pairs over 60 dies

In a charging event, it is expected that the weaker side will serve the dominant discharging path, leading to more prominent imbalance in the level of damages between left and right. Hence, only the devices with large *V*_BD_ difference between from the left and right sides are more likely to be resulting from plasma charging. Hence, the smaller *V*_BD_ in IMD breakdown on a pair is registered as the level correlated to PID stress level. Figure 4a shows three typical kinds of characteristics that are found on samples on different dies across a wafer. These samples can be categorized as no breakdown on either side, one-side breakdown or both-side breakdown groups. The portion of devices showing the particular characteristics of each group is shown in the pie chart of Fig. 4b. We found that on samples without antenna have much lower change of having one-side breakdown characteristics. Symmetric behaviors on both sides are found in most of the devices that has not experience plasma charging stress. These further suggest that most of the asymmetric characteristics in a differential pair come from charging stresses when antenna structure is attached.

**Fig. 5:**
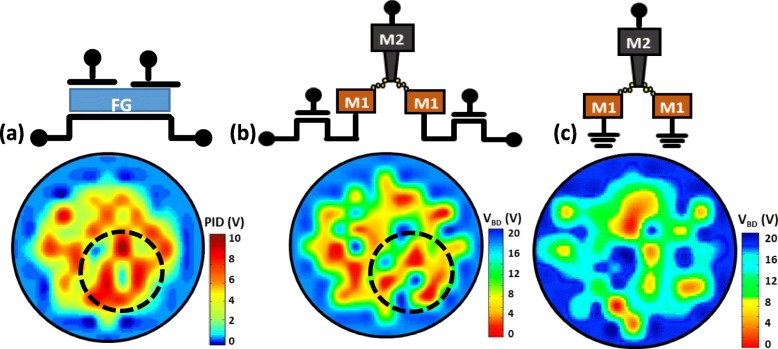
Schematics and the wafer maps showing the distributions of (**a**) the PID voltage and VBD from (**b**) test pattern I and (**c**) II, showing the regional effect within the circled area from plasma-induced damage on the back-end dielectric layer that can be attributed to the regional plasma charging levels

**Fig. 6:**
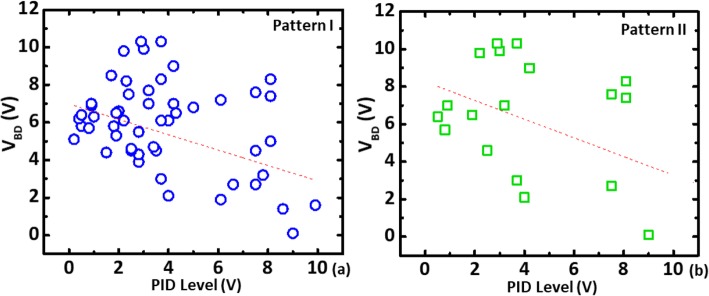
Correlations between VBD from PID levels for **a** pattern I and **b** pattern II samples, respectively [10–12]

**Fig. 7:**
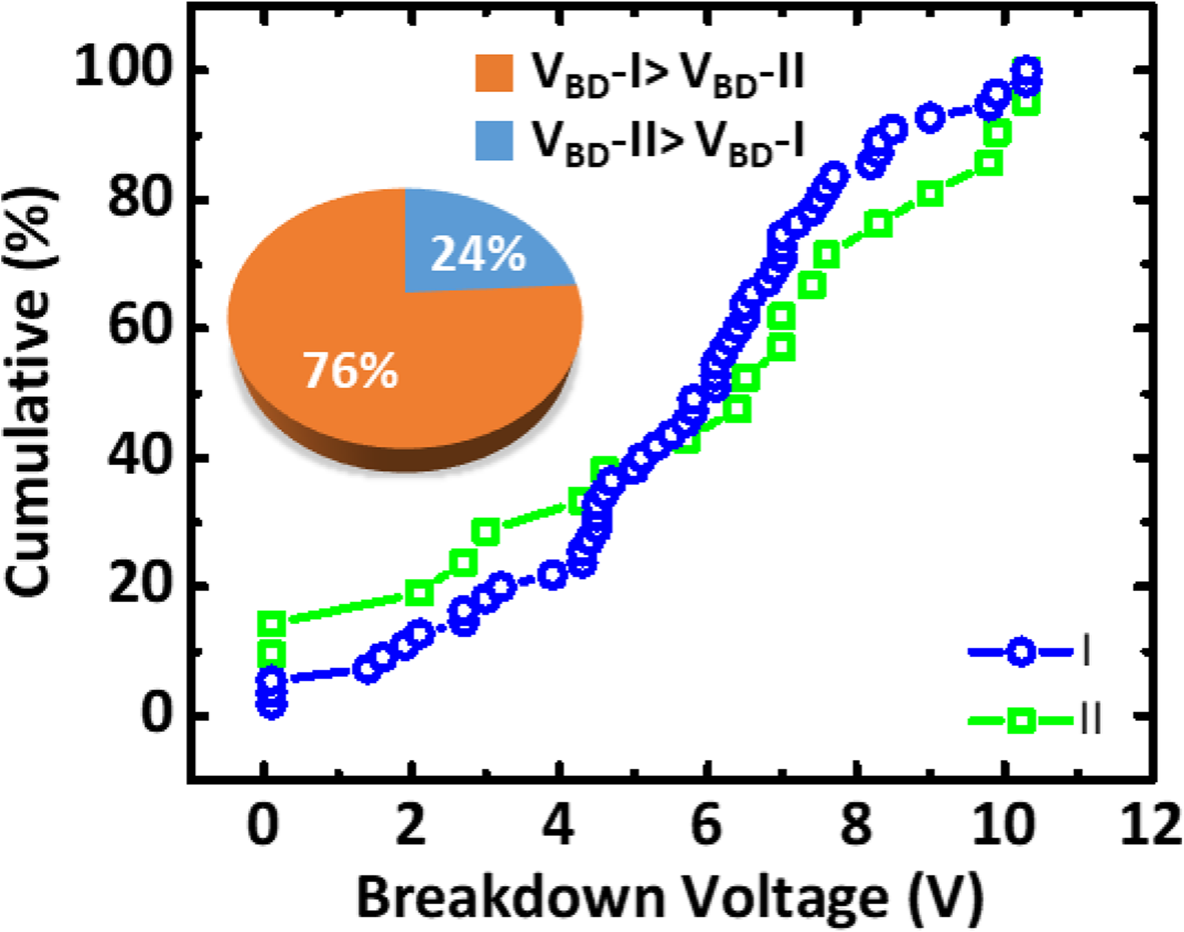
Comparison of cumulative distribution of *V*_BD_ from 60 dies across wafer and the portion of samples showing higher breakdown voltages from pattern I vs. that from pattern II

**Fig. 8:**
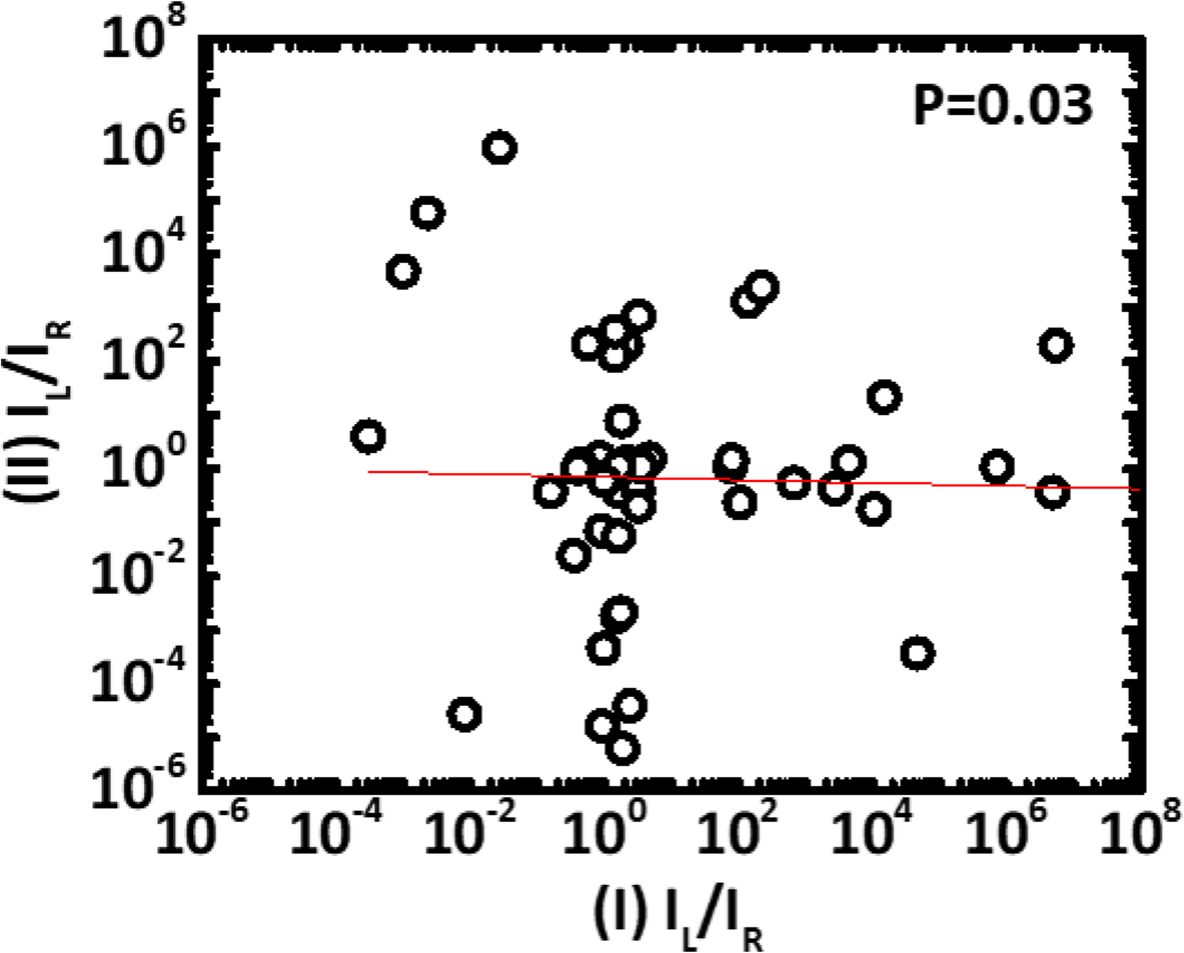
Leakage current ratio of left and right side of pattern I and II showing there is no systematic misalignment effect from these samples

**Fig. 9:**
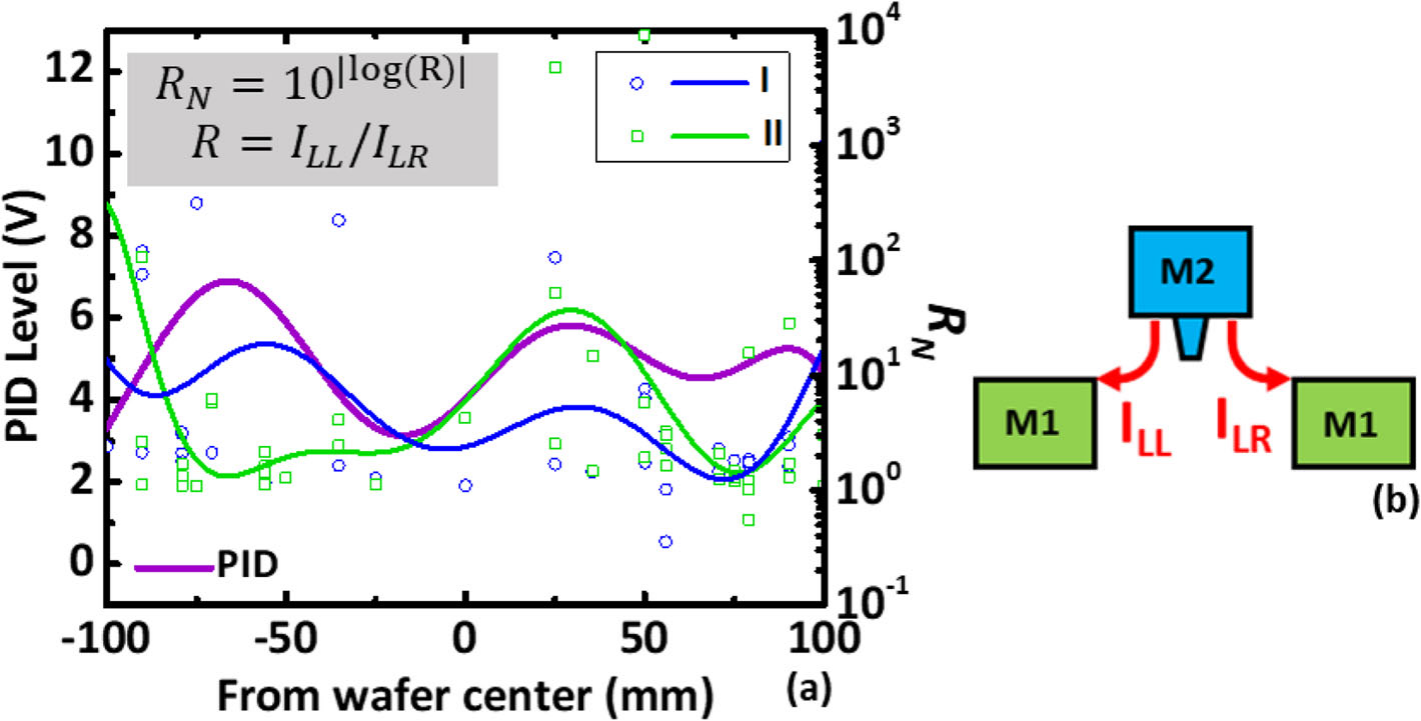
**a** Normalized current ratios from pattern I and II samples across wafer center as compared to the corresponding PID levels and **b** the definition of leakage current on the left (*I*_LL_) and right (*I*_LR_)

Using the *V*_BD_ obtained by the above method, the wafer maps of the PID level and *V*_BD_ from devices of type I and II patterns are compared in Fig. 5. High similarity between wafer maps of the *V*_BD_ from both type of monitoring devices and the corresponding reference charging levels is obtained from the PID recorder, see Fig. 5a. PID voltage is believed to record both electron and ion charging events during metal processes [14]. However, samples across a wafer are found to be a predominantly subject to more electron charging than ion charging [14]. We believe that some regional correlated can be seen between the wafer in Fig. 5a, b. While the wafer map in Fig. 5c from pattern II which no obvious similarity to that in Fig. 5a suggests that bi-directional stress [15] might lead to secondary effect on the dielectric layers, which need further investigation. Measured breakdown voltages from the new test patterns vs. the reference PID levels compared in Fig. 6 further reveal that the higher PID level on a die, the lower its *V*_BD_ the new test patterns. In addition, significant negative correlations between *V*_BD_ and plasma charging levels can be established. To investigate the effect of one-directional and bi-directional stresses on IMD damage, *V*_BD_ measured from devised with type I and type II patterns is summarized and compared in Fig. 7. The *V*_BD_ distribution obtained from devices on 60 dies across wafers indicates that the devices experienced bi-directional charging stresses are more likely to show IMD breakdown at a lower voltage. This could be explained by the asymmetric stress level as polarity changes across a IMD [16]. Moreover, stress-induced leakage current (SILC) measured with 10 V across the dielectric layer is another indicator for the increase of trap states [17] within the IMD film. To further minimized the die-to-die variation effect caused by process variation, the leakage ratio from each pair is used as the index to further evaluate the IMD damage. Figure 8 shown that there is essentially no correlation between the leakage current ratio on the two types of devices on the same die. That is to say that misalignment between metal 1 and via2 layers have minimal effects on both patterns. Noted that current ratio, *R*, defined as *I*_LR_/*I*_LL_, is a better index to remove noise from die-to-die variation on these patterns. On complete unbiased devices, the main charging stress occurs on the right or left that should be completely random. Here, some bias effects are found on the current ratios, where its distribution medium are not at 1. To ensure that only the damage results from the plasma charging effects are accounted for, *R* is the normalized current ratio, as described in Fig. 9. In the distribution plot across wafer center, *R*_N_ from devices of type I pattern follows with the plasma stress level from PID recorders fairly close. On the other hand, *R*_N_ from devices susceptible to bi-directional stress cannot be fully explained by the recorded PID levels. This might be caused by significant higher *R*_N_ level in devices of pattern II, as a result of addition ion charging events across wafer.

## Conclusions

Plasma-induced charging effect on the integrity of IMD films is investigated through newly proposed differential test patterns on advanced FinFET Cu BEOL platforms. Damages in the form of early breakdown and increase SILC in IMD layers are found to be directly correlated to the plasma charging levels across 12-inch wafers.

## Data Availability

Not applicable.
